# Prevalence of alcohol consumption and the associated factors among students at a Brazilian public university: a cross-sectional study

**DOI:** 10.1590/1516-3138.2023.0383.R1.03072024

**Published:** 2025-04-04

**Authors:** Lucas Carrara do Amaral, Lucas Lima Galvão, Douglas Assis Teles Santos, Gustavo Conti Teixeira Costa, Marilia Santos Andrade, Rodrigo Luiz Vancini, Katja Weiss, Beat Knechtle, Marciana Gonçalves Farinha, Claudio Andre Barbosa de Lira

**Affiliations:** I Human and Exercise Physiology Division, Faculty of Physical Education and Dance, Universidade Federal de Goiás (UFG), Goiânia (GO), Brazil.; II Department of Education, Physical Education College, Campus X, Universidade do Estado da Bahia (UNEB), Teixeira de Freitas (BA), Brazil.; III Professor, Department of Education, Physical Education Collegiate, Campus X, Universidade do Estado da Bahia (UNEB), Teixeira de Freitas (BA), Brazil.; IV Professor, Center for Advanced Study and Research in Sport, Faculty of Physical Education and Dance, Universidade Federal de Goiás (UFG), Goiânia (GO), Brazil.; V Professor, Departamento of Physiology, Escola Paulista de Medicina, Universidade Federal de São Paulo (UNIFESP), São Paulo (SP), Brazil.; VI Professor, Sports Department, Physical Education and Sports Center, Universidade Federal do Espírito Santo (UFES), Vitória (ES), Brazil.; VII Institute of Primary Care, University of Zurich, Zurich, Switzerland.; VIII Professor, Institute of Primary Care, University of Zurich, Zurich, Switzerland.; IX Professor, Institute of Psychology, Universidade Federal de Uberlândia (UFU), Uberlândia (MG), Brazil.; X Professor, Human and Exercise Physiology Division, Faculdade de Educação Física e Dança, Universidade Federal de Goiás, Goiânia (GO), Brazil.

**Keywords:** Alcohol drinking, Exercise, Social class, Education, graduate, Universities, Undergraduate students, Lifestyle, Physical activity, Alcoholism

## Abstract

**BACKGROUND:**

The World Health Organization estimated that approximately 43% of the global population consumes alcohol, with an average annual consumption of 4.6 L per person. However, little is known about the factors influencing alcohol intake among students.

**OBJECTIVES:**

This study aimed to determine the factors that influence alcohol intake in students at a Brazilian public institution.

**DESIGN AND SETTING:**

This cross-sectional study was conducted at a public university in the Brazilian Midwest.

**METHODS:**

In total, 348 Brazilian university students (124 men and 224 women; convenience sample) were recruited. The alcohol use disorder identification test (AUDIT) was used to examine alcohol use, the habitual physical activity questionnaire (Baecke) was used to assess physical activity levels, and the Brazil Economic Classification Standard Criterion was used to assess socioeconomic status. A generalized linear model (GLM) with a 95% confidence interval (CI) and odds ratio (OR) estimation was constructed using the Tweedie probability distribution and log link function, with AUDIT questionnaire scores as the dependent variable.

**RESULTS:**

The prevalence of excessive alcohol consumption was 18.7% (9.8% in men and 8.9% in women). The GLM analysis indicated that being single and attending an agricultural science course increased the likelihood of excessive alcohol intake; however, living with family or alone had a protective effect. Being single and pursuing a course in agricultural science increased the likelihood of binge drinking.

**CONCLUSION:**

Universities and families can use the study findings to develop initiatives aimed at enhancing students’ understanding of the harmful effects of alcohol, particularly among agricultural science students.

## INTRODUCTION

Harmful alcohol intake is associated with increased morbidity, mortality, and disability rates.^1^ It is linked to later menopause^2^ and an increased risk of cardiovascular, hepatic, and oncologic illnesses; pancreatitis and diabetes;^3,4^ and metabolic syndrome.^5^ Additionally, it increases the likelihood of memory loss, falls, traffic accidents, interpersonal conflicts, and unprotected sex.^6^ According to the National Alcohol and Drug Survey, the incidence of binge drinking increased by 31.1% from 2006 to 2012 (from 45% to 59% of the population).^7^ Consequently, alcohol intake accounts for 5.3% of all deaths worldwide.^1^

Alcohol is the most common risk factor among university students, who often overestimate its negative consequences, thereby increasing their risk exposure.^8^ Previous studies found that university students consume more alcohol than nonuniversity students, with a higher prevalence observed among male students^9,10^ Alemu et al.^11^ studied 741 university students at Jimma University in Ethiopia. They found that 26.5% of students had alcohol-related problems. Being single (odds ratio (OR) = 1.98, 95% confidence interval (CI) = 1.21–3.22), experiencing peer pressure to drink alcohol (OR = 2.72, 95%CI = 1.76–4.19), and having mental distress (OR = 2.81, 95%CI = 1.83–4.32) were independently and positively associated with alcohol use disorders.

According to Heather et al.,^12^ the prevalence of alcohol use varies significantly among colleges. In a binary logistic regression analysis of the effects of attending university, younger age, White ethnicity, and both on-campus and off-campus term-time student housing were identified as factors associated with alcohol use disorder, as measured using the Alcohol Use Disorders Identification Test (AUDIT). Furthermore, a study conducted in an Irish institution reported a 66.4% prevalence of excessive alcohol use. Students studying law and business were more than twice as likely to report harmful alcohol consumption compared with their peers studying science and engineering. This observation suggests that the field of study is a significant factor associated with increased alcohol consumption among male students (OR = 2.26; 95%CI = 1.46, 3.49; P < 0.001) and female students (OR = 2.12; 95%CI = 1.44, 3.14; P < 0.001). Alcohol intake has also been associated with poor self-rated health among university students.^13^ Additionally, student income can negatively affect alcohol consumption.^14^

The relationship between sports, exercise, and alcohol consumption is an ongoing area of research.^15^ Alcohol remains the most commonly used drug among athletes and regular exercisers, with alcohol-related issues appearing to be more prevalent in these groups.^16,17^ In human experimental research, alcohol consumption is categorized as acute (single dose) and chronic (repeated doses over time).^18^ According to this research, alcohol consumption impairs the utilization of glucose and amino acids by skeletal muscles, reducing energy supply and disrupting the metabolic processes during exercise.^18^ Furthermore, chronic alcohol consumption is associated with increased citrate synthase enzyme activity and decreased cross-sectional area of type I, IIa, and IIb fibers.^18,19^ Exercise appears to slow the ethanol-induced reduction in hepatic mitochondria and accelerate the liver’s ethanol metabolism.^18^ Additionally, regular training seems to reduce the extent of alcohol-induced oxidative damage.^18^

A study conducted by Wagner and Andrade^20^ in Brazil highlighted that the transition to university is a critical period of vulnerability for both initiation and continued use of alcohol and other drugs. Furthermore, alcohol misuse has been linked to an increase in depressive symptoms and a higher risk of suicide attempts.^21,22^ Learning difficulties, higher failure rates, decreased study commitment (e.g., reduced time spent studying and lower class attendance), and poor academic performance are some of the negative repercussions of heavy alcohol consumption in this population. Increased risk behaviors, such as increased frequency of unprotected sexual intercourse with different partners, sexual abuse, drunk driving, traffic accidents, public intoxication, physical altercations, violent deaths, and future problems with psychoactive substances (including dependence and the use of other psychoactive substances) have also been linked to heavy alcohol consumption.^23,24,25,26,27,28^ Given the limited research on factors influencing alcohol consumption among Brazilian university students, further investigation in this area is essential. This argument is further supported by the fact that factors related to excessive alcohol intake among university students vary owing to social, cultural, and economic differences. Therefore, gaining a deeper understanding of the factors related to alcohol consumption among Brazilian university students may reveal unique characteristics. Mapping these elements is crucial for establishing public policies aimed at reducing alcohol consumption among university students.

## OBJECTIVE

This study aimed to determine the factors influencing alcohol intake in a convenience sample of students at a Brazilian public university. We hypothesized that marital status, family circumstances, field of study, socioeconomic status, and physical activity would influence alcohol use among Brazilian college students.

## METHODS

### Participants

For this quantitative and cross-sectional study, 348 Brazilian university students (124 men and 224 women) from the Universidade Federal de Jataí (previously Universidade Federal de Goiás – Regional Jataí) were recruited. The participants were personally invited to the university and included undergraduate students and those aged > 18 years. Those who provided inaccurate responses to the surveys were excluded from the study. Before data collection, all participants were informed of the study methods and provided written informed consent. The study was approved by the Ethics Committee of the Universidade Federal Goiás (permission number: 124/13, date of approval: 11/04/2013) and adhered to the Declaration of Helsinki.

### Sociodemographic variables

A sociodemographic questionnaire was used to collect data on sex (male or female), age (years), undergraduate major (exact sciences, biological sciences, engineering, health sciences, agricultural sciences, linguistics, social sciences, and humanities), marital status (single or married), and place of residence.

### Alcohol consumption

The AUDIT^29,30^ was used to assess alcohol intake. This straightforward screening tool facilitates quick evaluations of excessive alcohol consumption.^31^ The AUDIT has been translated and validated in Brazilian Portuguese.^32^ The questionnaire comprises 10 items divided into three domains: (i) harmful alcohol use (frequency of consumption, typical quantity, and frequency of heavy drinking), (ii) dependence symptoms (impaired control over morning drinking and drinking), and (iii) harmful consequences of alcohol use (guilt after drinking, blackouts, alcohol-related injuries, and other alcohol-related concerns). The first eight items are scored on a scale of 0 to 4, while the last two items are scored as 0, 2, and 4.^32^ Scores of 0–7 indicate a “low-risk consumption,” 8–15 denote “overuse,” 16–19 suggest “possible dependence,” and 20–40 signify “clear dependence.” The AUDIT scores demonstrated good reliability, with a Cronbach’s alpha of 0.75, confirming its suitability for screening alcohol consumption problems in the university setting.^33^

### Habitual physical activity level

The level of habitual physical activity was assessed using a Brazilian Portuguese-translated and validated version of the Baecke questionnaire. The questionnaire contained 16 questions that assessed three categories of habitual physical activity over the past year: occupational physical activity (eight questions), leisure-time physical exercise (four questions), and sports physical activity (four questions). An overall score for habitual physical activity was derived by summing the scores from these three categories. Higher scores indicate frequent physical activity. The test-retest reliabilities of the work, sport, and leisure time indices were 0.88, 0.81, and 0.75, respectively.^34^

### Socioeconomic level

The Brazil Economic Classification Standard Criterion^35^ was used to assess socioeconomic status. This questionnaire comprised 10 questions divided into two domains: possession and education level of the head of the household. The responses are scored as follows: A1, 42–46; A2, 35–41; B1, 29–34; B2, 23–27; C1, 18–22; C2, 14–17; D, 8–13; and E, 0–7 points. Despite its widespread use, no study has evaluated its reliability.

### Statistical analysis

SPSS 23.0 (IBM Corp., Chicago, Illinois, USA) was used to perform all analyses. The Kolmogorov–Smirnov test was used to determine the normality of the data. The Mann–Whitney *U* test was used to compare AUDIT scores, age, occupational physical activity, sports physical activity, leisure-time physical activity, and socioeconomic status.

A generalized linear model (GLM) was developed using the Tweedie probability distribution and log link function with the AUDIT questionnaire scores used as dependent variables. The model included 95% confidence intervals (95%CI) and odds ratio (OR) estimates. Sex; marital status (single or married); age group (18–28 years old, 29–38 years old, and ≥ 39–48 years old); housing arrangements; field of study; socioeconomic status; and levels of occupational physical activity, sports physical activity, and leisure-time physical activity were used as independent variables. Univariate models were constructed sequentially, each involving one independent variable and one response variable, while all independent variables were included in the multivariate model. The model distributions were selected based on convergence parsimony and the lowest quality value according to the Akaike information criterion.^36^ Furthermore, the omnibus test was employed to confirm that the model outperformed the null hypothesis, (P < 0.05). The significance level was set at 5%.

## RESULTS

The study included 348 Brazilian university students with an average age of 22.87 ± 5.47 (18–57) years. The distribution of categorical variables by sex is presented in **Table 1**. Overall, the majority of participants (86.2%) were unmarried, lived with their families (66.4%), had an economic classification of B1/B2 (47.7%), and consumed alcohol (74.1%).

**Table 1:** Characteristics of the study participants according to sex

	Total	Men	Women
	n (%)	n (%)	n (%)
Marital status			
Single	300 (86.2)	115 (92.7)	185 (82.6)
Married	48 (13.8)	9 (7.3)	36 (17.4)
Family arrangement			
Alone	59 (17.0)	23 (18.5)	36 (16.1)
Family	231 (66.4)	71 (57.3)	160 (71.4)
Friends	58 (16.7)	30 (24.2)	38 (12.5)
Economic classification			
A1/A2	25 (7.2)	9 (7.3)	16 (7.1)
B1/B2	166 (47.7)	73 (58.9)	93 (41.5)
C1/C2	122 (35.1)	37 (29.8)	85 (37.9)
D/E	35 (10.1)	5 (4.0)	30 (13.4)
Alcohol consumption			
No	90 (25.9)	28 (22.6)	62 (27.7)
Yes	258 (74.1)	96 (77.4)	162 (72.3)
Field of undergraduate course			
Exact	35 (10.1)	25 (20.2)	10 (4.5)
Biological	15 (4.3)	3 (2.4)	12 (5.4)
Engineering	43 (12.4)	23 (18.5)	20 (8.9)
Health	67 (19.3)	8 (6.5)	59 (26.3)
Agrarian	55 (15.8)	30 (24.2)	25 (11.2)
Linguistics	12 (3.4)	6 (4.8)	6 (2.7)
Social	48 (13.8)	20 (16.1)	28 (12.5)
Human	73 (21.0)	9 (7.3)	64 (28.6)

Data are expressed as absolute (relative) frequency.

When alcohol intake was assessed, 65 (18.7%) participants reported consuming alcohol, comprising 34 (9.8%) men and 31 (8.9%) women.

A comparison of AUDIT scores of men and women, age, occupational physical activity, sports physical activity, leisure-time physical activity, and socioeconomic status is presented in **Table 2**.

**Table 2:** Characteristics of the participants according to sex

	Total	Men	Women	P
	(n = 348)	(n = 124)	(n = 224)	
AUDIT (score)	3.0 (6.0)	4.0 (8.0)	2.0 (5.0)	0.005
Age (years)	21.0 (4.0)	21.0 (3.0)	21.0 (5.0)	0.829
Occupational physical activity	2.2 (0.7)	2.2 (0.7)	2.3 (0.6)	0.287
Sports physical activity	2,0 (1.0)	2.5 (1.2)	2.0 (1.0)	< 0.001
Leisure-time physical activity	2,2 (1.0)	2.2 (1.0)	2.2 (1.0)	0.091
Socioeconomic level	23.0 (10.0)	25.0 (10.0)	22.0 (10.0)	0.001

Data are expressed as mean (standard deviation). AUDIT = alcohol use disorders identification test.

According to the multivariate GLM with Tweedie distribution, the risk of excessive alcohol consumption was 65% higher (OR = 1.65; 95%CI = 1.09–2.48) among single individuals than in married individuals and 64% higher (OR = 1.64; 95%CI = 1.10–2.44) in agricultural science students than in humanities students. Furthermore, students living alone or with family members had 32% (OR = 0.68; 95%CI = 0.48–0.95) and 33% (OR = 0.67; 95%CI = 0.51–0.88) less likely to engage in excessive alcohol intake, respectively (**Table 3**).

**Table 3:** A generalized model employing univariate and multivariate Tweedie distributions to analyze the relationship between independent variables and Alcohol Use Disorders Identification Test scores

Variables	%	AUDIT			
		Univariate		Multivariate*	
		OR (95%CI)	P	OR (95%CI)	P
Marital status			< 0.001		0.016
Single	86.2	2.61 (1.82–3.74)		1.65 (1.09–2.48)	
Married	13.8	1		1	
Family arrangement			< 0.001		0.010
Alone	17.0	0.63 (0.45–0.89)		0.68 (0.48–0.95)	
Family	66.4	0.59 (0.45–0.77)		0.67 (0.51–0.88)	
Friends	16.7	1		1	
Field of study			0.002		0.014
Exact	10.1	1.24 (0.79–1.96)		0.81 (0.50–1.31)	
Biological	4.3	1.36 (0.79–2.34)		1.16 (0.67–2.03)	
Engineering	12.4	1.80 (1.14–2.85)		1.33 (0.86–2.04)	
Health	19.3	1.34 (0.87–2.09)		1.15 (0.77–1.72)	
Agricultural	15.8	2.28 (1.55–3.36)		1.64 (1.10–2.44)	
Linguistics	3.4	0.73 (0.32–1.66)		0.77 (0.34–1.74)	
Social	13.8	1.93 (1.29–2.89)		1.47 (0.99–2.19)	
Human	21.0	1		1	
Economic classification			0.001		0.009
A1/A2	7.2	1.39 (0.81–2.36)		1.39 (0.82–2.36)	
B1/B2	47.7	1.03 (0.69–1.53)		0.96 (0.67–1.40)	
C1/C2	35.1	0.65 (0.43–0.98)		0.69 (0.47–1.01)	
D/E	10.1	1		1	
Occupational physical activity		1.11 (0.94–1.32)	0.198	1.10 (0.94–1.29)	0.203
Sports physical activity		1.15 (0.99–1.35)	0.064	1.01 (0.85–1.20)	0.855
Leisure-time physical activity		1.12 (0.96–1.32)	0.138	1.06 (0.89–1.27)	0.465

*Adjusted for sex and age. AUDIT = alcohol use disorders identification test; OR = odds ratio; CI = confidence interval.

## DISCUSSION

This study aimed to determine the factors associated with alcohol use among the students of a Brazilian public institution. The study findings indicated that marital status, living arrangements, study area, and socioeconomic status were associated with excessive alcohol use, thus confirming our initial hypothesis.

In terms of marital status, being single increased the likelihood of excessive alcohol consumption by 65% compared with being married. Living with family and alone reduced the likelihood of excessive alcohol consumption by 33% and 32%, respectively, compared with living with friends. Additionally, students majoring in agricultural sciences were 64% more likely to engage in excessive alcohol use than those with other majors, and a higher socioeconomic status significantly increased the risk of excessive alcohol consumption.

Previous research has established a link between increased alcohol use and academic settings. For example, more than 66% of United Kingdom university students reported excessive alcohol intake.^37^ Our findings align with those of Santos et al.^38^, who identified a 24% prevalence in a sample of 1,290 Brazilian university students of both sexes. In comparison, a study of Colombian university students reported a prevalence rate of 20.5%.^39^ Similarly, the prevalence rate among students in the United States was 18%.^40^ In addition, a study conducted in Morocco showed an 8.5% prevalence in a sample of 1,236 students of both sexes.^41^ The difference in alcohol consumption prevalence is attributed to the different tools used to evaluate alcohol use; the influence of local public laws that control alcohol use; and cultural, economic, social, and religious aspects.^24^ For example, compared with other regions worldwide, Muslim countries have lower prevalence rates of excessive alcohol intake among young people aged 15–19 years; their low ranking may be attributable to the religious prohibition against consuming alcoholic beverages.^41^ The high alcohol consumption rate observed among our participants may be related to the experience of being a university student, as it often provides individuals an initial opportunity to be part of a large group of peers without familial supervision.^42^ Consequently, individuals may become more inclined to try new things, such as alcohol consumption.

In our study, the prevalence of excessive alcohol consumption was 9.8% in men and 8.9% in women, with no significant difference in alcohol use between the sexes. However, other studies have found a link between sex and alcohol use.^38,43,44,45^ Sociocultural factors may account for these variations in alcohol intake between men and women. Although men are twice as likely as women to abuse alcohol, the severity and types of alcohol-related issues are often similar.^46^ The factors contributing to our findings could include a shift in women’s societal roles and a trend toward gender equality, in which women invest more in education, work outside the home, adopt behaviors traditionally associated with men, and consume more alcohol, thereby reducing disparities in the consequences of alcohol use.^46^ Furthermore, women’s increased alcohol consumption may be influenced by stress from balancing work and domestic responsibilities.^46^

A few studies have examined the effect of marital status on the prevalence of excessive alcohol consumption. In the present study, marital status emerged as a significant factor in the multivariate model, revealing that single individuals were 65% more likely to drink excessively than married people. Similar findings have been reported in other studies.^44,47,48,49,50^ In Sweden, students in committed relationships, whether dating or married, were less likely to consume large amounts of alcohol and exhibited lower AUDIT scores compared with their single peers.^51^ In Finland, marital status did not influence the prevalence of alcohol consumption among students, although only married male students consumed less alcohol than unmarried counterparts. In Italy, no significant relationship was found between marital status and excessive alcohol consumption.

Several studies have explored the relationship between alcohol consumption and living arrangements, such as living with friends, family, or alone. These studies found that students who lived alone, in student housing, or with roommates consumed more alcohol or had higher rates of binge drinking compared with those who lived with their parents, a partner, and/or children.^52^ According to the Cutting down, Annoyance by criticism, Guilty feeling, and Eye openers (CAGE) screening instrument (another instrument for assessing alcohol consumption),^52^ students living with their families drink alcohol more frequently, but report fewer problems related to alcohol abuse. Kuntsche et al.^53^ found that social drinkers consumed alcohol more frequently at mixed parties but not at home, in bars, or with family members. Enhancement drinkers drank with same-sex friends at home, with friends, and in bars. By contrast, coping drinkers drank at home but not at parties or with family members.^53^ The factors influencing alcohol consumption include the tendency to take risks and test limits, the tendency to seek new and potentially dangerous situations, general impulsivity typical of young people, the desire for acceptance by peers (which are environmental factors that influence the development of the habit of drinking and the reference of parents and family),^54^ and having a positive/pleasant experience (e.g., being more communicative, having more success in looking for partners, and having more fun).^54^

The patterns of alcohol and drug use vary depending on the academic field of students; however, no systematic research has explored this topic.^52^ All studies indicated significant variations between study locations; however, no apparent pattern was found.^52^ Specific interests, the relationship between academic knowledge with behavior, varying workloads, gender distribution, ethnic diversity, and physical activities can influence alcohol and drug consumption patterns.^52^ Alcohol consumption among medical students is of particular concern, as their perspectives on alcohol may shape their future clinical practice, especially when treating patients with alcohol-related issues.^52^ Country music is one of the most prevalent cultural aspects in the daily lives of the Brazilian people. It frequently features lyrics that encourage alcohol consumption. This influence is particularly significant among university students, who are at higher risk of excessive alcohol consumption.^54^ This may explain why agricultural undergraduate students reported higher levels of alcohol consumption. Furthermore, UFG-Jataí is located in a rural community with a thriving agricultural industry.

With regard to the current state of alcohol consumption in Brazil, the World Health Organization Global Report on Alcohol and Health 2018 indicated an 11% decrease in alcohol consumption per capita in the country over 6 years, dropping from 8.8 L in 2010 to 7.8 L in 2016. In the same period, the prevalence of alcohol use disorders also declined (from 5.6% to 4.2%).^1,55^ These results were influenced by the implementation of the prohibition law no. 11,705/2008, along with its subsequent intensification (in 2012 and 2016) and law no. 13,106/2015, which criminalized the provision of alcohol to individuals below 18 years old.^1,55^ However, several challenges persist, including the need for further research on prevention and treatment to reduce the prevalence of harmful alcohol use. Other key areas of focus include reducing average daily consumption to levels comparable to those in the Americas, decreasing the frequency of binge drinking, preventing early alcohol experimentation among children and adolescents, and lowering the incidence of alcohol-related hospitalizations among the elderly population.^1,55^

In Sweden, web-based screening and brief interventions have been proven to be practicable, acceptable to students, and effective in reducing drinking risks for 6–12 months.^51^ Similarly, a study conducted by Kypri et al.^56^ demonstrated that brief interventions delivered in primary care settings could prevent excessive alcohol intake. This research aimed to assess the young people’s acceptance of screening offers in primary care settings, identify their alcohol consumption levels, and estimate the proportion of patients who would benefit from a brief virtual intervention and follow-up. This study shows that primary care settings can effectively facilitate remote access to a large number of people who consume excessive amounts of alcohol. In another study, two 24-h sessions of motivational intervention and psychoeducation significantly reduced alcohol consumption in 12 months among students whose parents had alcohol issues.^57^ Physical exercise also emerged as a highly effective intervention for lowering the consumption of several addictive substances. It promotes dopamine, glutamate, and endogenous opioid pathways, which help lower acute cravings, enhance the experience of pleasure, regulate mood, and alleviate the symptoms of depression and anxiety.^58^ These insights underscore the importance of providing university students with tools to identify and address the risk factors and implementing practical and cost-effective interventions to reduce hazardous alcohol use.

The current study has some limitations. First, like other questionnaire-based studies, the accuracy of these results relies on the respondents’ honesty and memory. Second, potential confounding factors, such as a family history of alcoholism, were not assessed. Third, the cross-sectional design of the study limits our ability to infer causality. Despite these limitations, the findings remain significant and contribute valuable insights.

## CONCLUSION

In conclusion, the study population showed a high prevalence of excessive alcohol consumption. Factors such as being unmarried, living with friends, participating in undergraduate agricultural sciences programs, and having a good socioeconomic status were associated with excessive alcohol intake. Identifying these factors is crucial for formulating public policies aimed at reducing alcohol use among university students. Future longitudinal studies should prioritize interventions aimed at reducing alcohol consumption among university students.
